# The Effect of Lipopolysaccharides from *Salmonella enterica* on the Size, Density, and Compressibility of Phospholipid Vesicles

**DOI:** 10.3390/biomimetics10010055

**Published:** 2025-01-15

**Authors:** Tamás Szabó, Zuzana Garaiová, Sopio Melikishvili, Marek Tatarko, Zsófia Keresztes, Tibor Hianik

**Affiliations:** 1 Functional Interfaces Research Group, Institute of Materials and Environmental Chemistry, HUN-REN Research Centre for Natural Sciences, Magyar Tudósok Körútja 2, 1117 Budapest, Hungary; keresztes.zsofia@ttk.hu; 2 Department of Nuclear Physics and Biophysics, Faculty of Mathematics, Physics and Informatics, Comenius University, Mlynska Dolina F1, 84248 Bratislava, Slovakia; zuzana.garaiova@fmph.uniba.sk (Z.G.); marek.tatarko@fmph.uniba.sk (M.T.); 3 Institute of Medical Physics and Biophysics, Faculty of Medicine, Comenius University, Sasinkova 2, 81372 Bratislava, Slovakia; sopio.melikishvili@fmed.uniba.sk

**Keywords:** phospholipid vesicles, lipopolysaccharides, average size, zeta potential, density, ultrasound velocity, attenuation, adiabatic compressibility

## Abstract

The properties of the large unilamellar vesicles (LUVs) from 1,2-dimyristoyl-sn-glycero-3-phosphatidylcholine (DMPC), modified by lipopolysaccharides (LPS) from *Salmonella enterica* sv. Enteritidis, which mimics Gram-negative bacteria, were studied by various physical methods. LPS, in the range of 0/20/50 % *w*/*w* relative to the lipid, had a regulatory role in the structure of the LUVs toward the lower size, low polydispersity, and over-a-month size stability due to the lower negative zeta potential. The addition of LPS resulted in increased density, which determined the ultrasound velocity and the specific adiabatic compressibility. In a 0.5/1/2 mg/mL concentration range, the total lipid content did not significantly affect the size of LUVs and influenced the density-related attributes similarly to the LPS content. A positive correlation was found between temperature and vesicle size, and a negative correlation was found between temperature and density and compressibility—except for the anomaly behavior at 25 °C, around the melting point of DMPC.

## 1. Introduction

*Salmonella enterica* (*S. enterica*) is a pathogenic bacterium responsible for 41.9% of human Salmonella infections, with increasing concerns about antimicrobial resistance [1,2]. These findings motivate biomimetic research into the infection mechanisms, as well as the early detection and potential prevention of *S. enterica* outbreaks [3]. The bacterial membrane plays a key role in cell invasion of the host organization. In Gram-negative bacteria like *S. enterica*, lipopolysaccharides (LPS) in the outer membrane, particularly lipid A, are key components responsible for LPS’s toxic effects through interactions with host immune cell membranes [4]. Both bacterial and host cell membranes consist of phospholipids, which highly contribute to membrane structure and function.

Because of the complexity of biomembranes, simplified membrane models like liposomes (dispersed in buffer), lipid monolayers (at air–water interfaces), or supported lipid bilayers (SLBs, transferred to solid surfaces) are often used as host media for LPS, to understand the roles of membrane features in lipid–LPS interactions [5,6,7,8]. These models can give a basis to vaccine adjuvants [9,10] and therapeutic and diagnostic applications by engineering biosensor platforms that harness the specific interactions between LPS and antibodies or, concerning antimicrobial resistance, between LPS and antibiotics [6,11].

Due to the unique properties of LPS, its incorporation into an artificial membrane is a challenging task. Several strategies, including the inverted emulsion method and the hydration method followed by extrusion or sonication, have been developed to incorporate LPS into bilayers [12,13,14]. Although it has been reported that *S. enterica.* LPS has a preference for insertion into mixed and cholesterol-rich membranes [15], pure 1,2-dimyristoyl-sn-glycero-3-phosphocholine (DMPC) is a popular synthetic lipid for membrane models for its neutral overall charge and low main transition temperature (around 24 °C), enabling convenient liposome formation and surface adhesion at room temperature [13,14].

Small unilamellar liposomes were used to develop SLBs made of DMPC lipids, LPS, and outer membrane proteins extracted from *E. coli*, and their atomic force microscope study showed that LPS can incorporate into a DMPC bilayer, forming rough domains and bump-like aggregates [13]. Multilamellar DMPC vesicles were also used as hosts for *E. coli* LPS to study its influence on the structure, dynamics, and mechanical strength of phospholipid membranes [14], and it was shown that the amplitude of collective tumbling motions of DMPC molecules was increased by LPS, resulting in decreased vesicle size under shear conditions. The destabilization and shape-alteration effect of *E. coli* LPS on lipid bilayer was shown in giant unilamellar vesicles as well [16].

To the best of our knowledge, there is a gap in the literature regarding *S. enterica* LPS-containing vesicle model membranes. Most scholars have studied LPS from *E. Coli*, incorporated into small or giant unilamellar vesicles or SBLs. Nevertheless, their results encouraged us to continue the exploration of detailed biophysical interactions between LPS from *S. enterica* and DMPC, blended in large unilamellar vesicle systems. Such systems can form part of sensing platforms, i.e., vesicles, or monolayers and SBLs, which may undergo structural changes, i.e., aggregation or weight increase, upon exposure to LPS-specific antibodies, aptamers, or antibiotics as analytes.

In this study, we focus on the detailed investigation of the effects of LPS from *S. enterica* and total lipid content on vesicle size, polydispersity, stability, zeta potential, density, and specific adiabatic compressibility at varying temperatures. We use dynamic light scattering for monitoring Brownian and electrophoretic particle movement; densitometry; and ultrasound spectroscopy. The results could contribute to a better understanding of the influence of LPS on phospholipid membranes and guide the optimization of LPS-liposomal platforms for biosensing applications. For the first time, we applied precise density measurements and ultrasound spectroscopy to analyze the adiabatic compressibility of the vesicles with incorporated LPS.

## 2. Materials and Methods

### 2.1. Materials

Deionized water with a resistance of 18.2 MΩ.cm has been prepared by Purelab Classic UV (Elga, High Wycombe, UK) and used in all experiments. The large unilamellar vesicles (LUV) have been prepared from 1,2-dimyristoyl-sn-glycero-3-phosphatidylcholine (DMPC) (Sigma-Aldrich, Darmstadt, Germany, Cat. No. P2663) and contained various weight ratios of lipopolysaccharides (LPS) isolated from *S. enterica* serotype Enteritidis (Sigma-Aldrich, Cat. No. L6011). A 20 mM TRIS buffer containing 70 mM NaCl (pH 7.4) has been filtrated through a 0.22 µm syringe filter Milex^®^ MCE (Merck-Millipore, Darmstadt, Germany, Cat. No. SLGSVR255F) and has been used for preparation of LUV using a mini extruder based on the polycarbonate membranes with a 100 nm pore size (Avanti Polar Lipids, Inc., Birmingham, AL, USA) according to the method of MacDonald et al. [17]. All standard chemicals were purchased either from Sigma-Aldrich or Slavus (Bratislava, Slovakia) and used as supplied without additional purification.

### 2.2. Preparation of LPS-DMPC Vesicles

The LUVs prepared from DMPC with 0, 5, 20, and 50 *w*/*w*% LPS content (percentage of the total lipid content LPS + DMPC) were prepared based on the procedure of Kiss et al. [13] via the extrusion technique mentioned above. As a reference to certain investigations, a solution of pure LPS (100% LPS content) was prepared as well. The total lipid concentrations of the final samples were 0.5, 1, and 2 mg/mL. First, the DMPC was dissolved in chloroform at a concentration of 5 mg/mL and was transferred to a round-bottomed flask, and the chloroform was slowly removed with nitrogen flushing, during continuous turning of the flask in such a way that the DMPC was dried onto the lower third part of the inner side of the flask. Then, the corresponding amount of LPS dissolved in a TRIS buffer was added to achieve the total concentrations of lipid (c_total_) of 0.5, 1, and 2 mg/mL. Hydration of such DMPC-LPS-containing buffer was performed at 40 °C. This temperature was maintained throughout the whole procedure, including 1 h of intense 500 rpm shaking of the re-hydrated sample and its batch-wise extrusion through a 100 nm pore-size polycarbonate membrane. The extrusion was carried out by performing 21 extrusions with the syringes installed in the mini extruder. These hydrated + extruded samples are identified in the study as ‘Extruded’. To observe the role of extrusion, samples without the extrusion step were also prepared and are referred to throughout the text as ‘Hydrated’. The samples, both extruded and hydrated, were kept at 4 °C for long-term storage. Before every measurement, the samples were left to adjust to the room temperature and were shaken by a pipette or vortex mixer.

### 2.3. Average Size and Polydispersity Measurement

The zeta-average size (Z-Avg or cumulant size) and polydispersity index (PDI) of the vesicles were measured with the dynamic light scattering (DLS) technique with a Zetasizer Nano ZS90 (Malvern, UK), with the following settings: medium: 20 mM TRIS buffer containing 70 mM NaCl, pH 7.4; temperature: 20, 25, and 30 °C; detector position: 90°; conditioning time: 120 s; 3 measurements with automatically set attenuation and number of runs; general purpose measurement mode and Smoluchowski data fitting was applied. The samples were held in disposable DS1600 plastic cuvettes. The results were averaged from the triplet measurements, where mainly intensity-weighted (cumulant fit) data were evaluated. Volume- and number-related size distributions were considered as well.

### 2.4. Zeta Potential Measurement

Zeta potential measurements were realized with the same Malvern instrument (see Section 2.3), using the default Malvern dip cell for aqueous samples. The settings were the same as in the case of the Z-Avg/PDI measurements.

### 2.5. Absorbance and Fluorescence Measurements

Absorbance and fluorescence spectra of the LPS-DMPC vesicles were recorded with a Varioskan Flash plate reader (Thermo Fisher Scientific, Vienna, Austria, data collection: SkanIt, ver. 2.4.5, default software of the instrument), at 25 °C. The clarity of the samples was measured from the histogram of the samples’ photographs. Pictures were taken of the samples pipetted in the optical plates, and the color (white) coordinate was extracted from their greyscale version. Picture management was carried out with the software IrfanView ver. 4.54.

### 2.6. Density Measurement

Density values of LPS-DMPC samples were measured with a laboratory density meter DMA 1001 (Anton Paar, Graz, Austria) equipped with a U-shaped tube to provide a fixed sample volume of 2 mL. The samples were degassed by mechanical agitation while being placed into a vacuumed syringe. All samples were measured at 20, 25, and 30 °C. The results were averaged from triplet measurements. Re-calibration of the device was performed before changing temperatures, and the calibration was checked frequently by measuring the density of water and air.

### 2.7. High-Resolution Ultrasound Velocimetry Measurement

A high-resolution ultrasound spectrometry (HRUS) technique with HR-US 102 (Sonas Technologies, Dublin, Ireland) ultrasound equipment was used to measure simultaneously the velocity and attenuation of ultrasound passing through the LPS-DMPC vesicles. These ultrasound parameters help to understand interrelated characteristics such as density, compressibility, etc. [18,19,20]. Here, the HRUS method was not used as literal spectrometry, i.e., scanning through a certain frequency range and measuring the velocity/attenuation of the sound, but as velocimetry, only to obtain velocity and attenuation values at given frequencies, at which the instrument was continuously operated. The operational frequencies were 2760, 5144, 8148, and 15,035 kHz, chosen for measurement peak Nr. 1, 2, 3, and 4, respectively. The samples were degassed by mechanical agitation while being placed into a vacuumed syringe. All samples were measured at 20, 25, and 30 °C for 30–50 min to establish stabilized data.

### 2.8. Specific Adiabatic Compressibility Calculations

Specific adiabatic compressibility (φ_K_/β_0_) represents the changes in the compressibility of the vesicles, relative to the solvent. The addition of various compounds to lipoid vesicles causes alterations in the basic structure, depending on which part of the bilayer and to what extent the added compound can be dispersed. With good miscibility, the additional molecule slips in between the hydrophobic chains or the hydrophilic headgroups (or both) of the lipid molecules and induces a thermodynamic disorder, which is, in the end, realized in increased fluidity and lower transition temperatures [21]. However, the compactness of the vesicles can be affected by the added component in various ways. There are studies reporting various ‘effectors’ that increased compactness and thus lowered compressibility [21,22,23,24], while free volume and compressibility can be increased as well [25,26,27]. Additionally, the effect of the same molecule can be different depending on whether the solid gel or the liquid crystalline phase of the vesicles is considered [28,29,30]. The compressibility of LPS-DMPC samples was calculated based on the study of Halstenberg et al. [28] and Melikishvili et al. [22], using the measured density and ultrasound velocity data.

The calculations were completed as follows:

(*u* − *u*_0_)/*u* ≈ (*f* − *f*_0_)/*f* (1)

where u is the velocity of ultrasound in the sample, *u*_0_ is the velocity in the solvent, *f* is the frequency of ultrasound in the sample, and *f*_0_ is the frequency in the solvent.

[*u*] = (*u* − *u*_0_)/(*cu*_0_) (2)

where [*u*] is the velocity number in mL/g, and *c* is the concentration of the dissolved material in g/mL.

*φ_V_* = [1 − (*ρ* − *ρ*_0_)/*c*]/*ρ*_0_ = 1/*ρ*_0_ − [*ρ*] (3)

where *φ_V_* is the partial specific volume, *ρ* is the density of the sample in g/mL, and *ρ*_0_ is the density of the solvent in g/mL.

[*ρ*] = (*ρ* − *ρ*_0_)/(*cρ*_0_) (4)

where [*ρ*] is the density number in mL/g.

*φ_K_*/*β*_0_ = −2 [*u*] − 1/*ρ*_0_ + 2*φ_V_* (5)

where *φ_K_*/*β*_0_ is the specific adiabatic compressibility in mL/g, and *β*_0_ is the adiabatic compressibility coefficient.

## 3. Results and Discussion

### 3.1. Average Size and Polydispersity of the Vesicles

**Figure 1:**
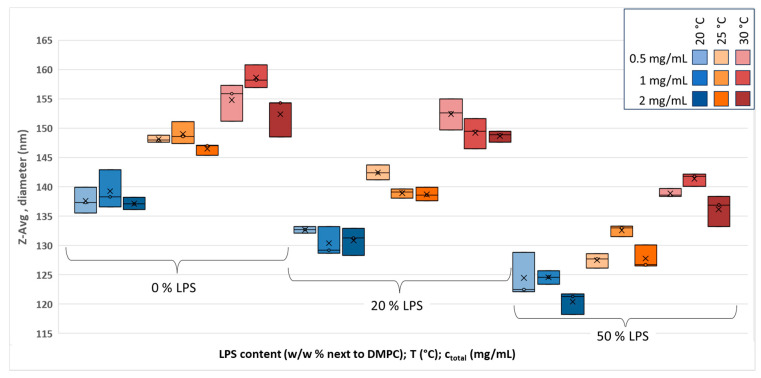
Z-Avg size variation in extruded LPS-DMPC vesicles with temperature (20–30 °C), LPS concentration (0–50 *w*/*w*%), and total lipid concentration (0.5–2 mg/mL) values.

The dependence of the size (Z-Avg, diameter) of hydrated and extruded LPS-DMPC vesicles on the LPS content (*w*/*w*%), temperature, and total lipid concentration (c_total_, mg/mL) was investigated with the DLS method, considering the intensity-related total cumulants fit. The results for extruded vesicles are shown in Figure 1. The data can be divided into three large groups that differ by LPS concentration. Each group consists of three subgroups, which differ in the measurement temperature. The subgroups contain triplets of samples with different c_total_ values.

The integration of LPS causes an overall diameter decrease for every subgroup. This is probably due to the steric repulsion between the negatively charged LPS incorporated into the vesicles, which shortens the radius of the spherical curvature. This phenomenon was reported by Sriwongsitanont et al. with distearoylphosphatidylethanolamine-PEG2000 added to DMPC vesicles and was explained with the steric hindrance of strongly hydrated PEG [31]; however, a simultaneous effect of steric and charge-related repulsion in PEG–lipid systems was also considered [32]. With 50% LPS added, the average diameter of the vesicles decreases by about 15 nm. On the other hand, the temperature increasing from 20 °C to 30 °C results in an average of a 20 nm increase in the diameter, independent of the LUV composition, which refers to thermal expansion through increased molecular mobility. As can be concluded from the subgroups, with respect to the LUV concentration, when increased to 2 mg/mL, the size decreases by a few nanometers, although it does not follow a clear trend when the 1 mg/mL samples are also considered. A possible explanation of the decreased sizes of the c_total_ = 2 mg/mL samples can be explained by increased steric pressure (charge-related and/or steric) on each other. However, this suggestion does not explain why, at c_total_ = 1 mg/mL, the LUV does not show a size decrease.

The polydispersity index (PDI) is the indicator of the narrowness of the size distribution. It is calculated automatically by the software from the intensity data as well (PDI = [(St. dev^2^)/(Mean^2^]). As shown in Figure S1 (see the Supplementary Materials), the PDI values for the extruded 0% and 20% LPS samples are mostly below 0.1, which is evidence of a relatively narrow size distribution. This value is approached and exceeded somewhat only with 50% LPS. Temperature and c_total_ do not have significant effects on the PDI, except for the highest 2 mg/mL total lipid concentration of the 50% LPS-containing samples.

**Figure 2:**
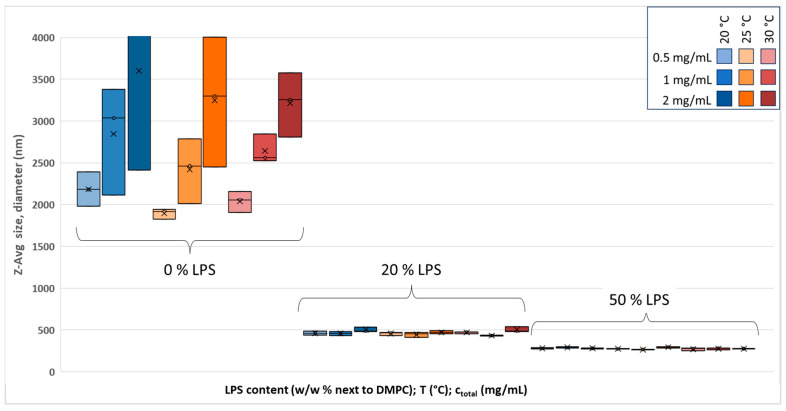
Z-Avg size variation in hydrated DMPC-LPS vesicles with temperature (20–30 °C), LPS concentration (0–50 *w*/*w*%), and total lipid concentration (0.5–2 mg/mL) values.

We also measured the average size of hydrated vesicles. The results are presented in Figure 2.

In Figure 2, very high Z-Avg values (up to 4000 nm) of the hydrated vesicles can be seen. For 0% LPS, the deviations are huge, which agrees with the expectations; i.e., the preparation of uniform vesicles needs the extrusion step to lose the irregular size. The size-altering effect is also represented in Figure S3a (see the Supplementary Materials) with size distribution spectra. However, from Figure 2, the addition of LPS seems to have a regulatory effect, as 20% and 50% LPS-DMPC vesicles show ~500 nm and ~300 nm Z-Avg sizes. PDI data in Figure S2 related to the hydrated vesicles also confirm that the pure DMPC vesicles are completely irregular, and although the PDI for 20% and 50% LPS-DMPC vesicles (~0.6) are well above the qualifiable level, the PDI deviation for these vesicles is smaller.

The discussion of the hydrated samples requires some additional notes. The Z-Avg and PDI data shown in Figure 2 and Figure S2, respectively, were derived from intensity data, which need some extended negotiation. When obtaining the Z-Avg size, the software simply gives the average position of all the peaks found on the intensity-related size distribution spectra. The general difference between the Z-Avg size distribution of extruded and hydrated samples is that the size distribution of extruded 20% and 50% LPS samples had only one distribution peak (Figure S3c,d, light red lines), which referred to the LPS-DMPC vesicles. Therefore, the values presented in Figure 1 and Figure S1 refer to the one exact peak and can be accepted as they are. Meanwhile, the hydrated samples with the same 20% and 50% LPS had at least two different peaks (Figure S3c,d, light-blue dashed lines). A peak representing a smaller-sized fraction appears at ~100 nm, and the larger one(s) refer to micron- or above-micron-sized structures. In Figure S3b, it was shown that LPS can form particle-like structures below 100 nm, and some studies refer to LPS ‘aggregates and vesicles around 100–200 nm’ [13,33,34,35]. These ~100 nm populations of hydrated samples will be discussed as LPS or LPS-rich structures/vesicles. From these peaks, the software automatically calculates Z-Average sizes, which are, therefore, somewhere between the lowest and highest sizes: ~500 nm and ~300 nm for 20% and 50% LPS, respectively (Figure 2).

A further interpretation is given with the analysis of intensity-, volume-, and number-related size distribution DLS spectra to resolve the here-presented hydrated data (Figure 2, Z-Avg, and Figure S2, PDI). The detailed analysis of the size distribution of the sample studied is presented in part S2 of the Supplementary Materials.

### 3.2. Average Size and PDI Stability of the Vesicles

The size and PDI-related stability of the extruded samples were monitored for almost two months. Here, we report about a 35-day interval. Throughout the investigating period, the samples were stored at ~4 °C, but for the measurements, they occasionally spent a few hours at 25 °C, i.e., room temperature. Before the DLS measurement, the samples were re-dispersed with a vortex mixer, because even the extruded and LPS-containing samples suffered a little sedimentation, reversely proportional to the LPS content, although they did not aggregate.

**Figure 3:**
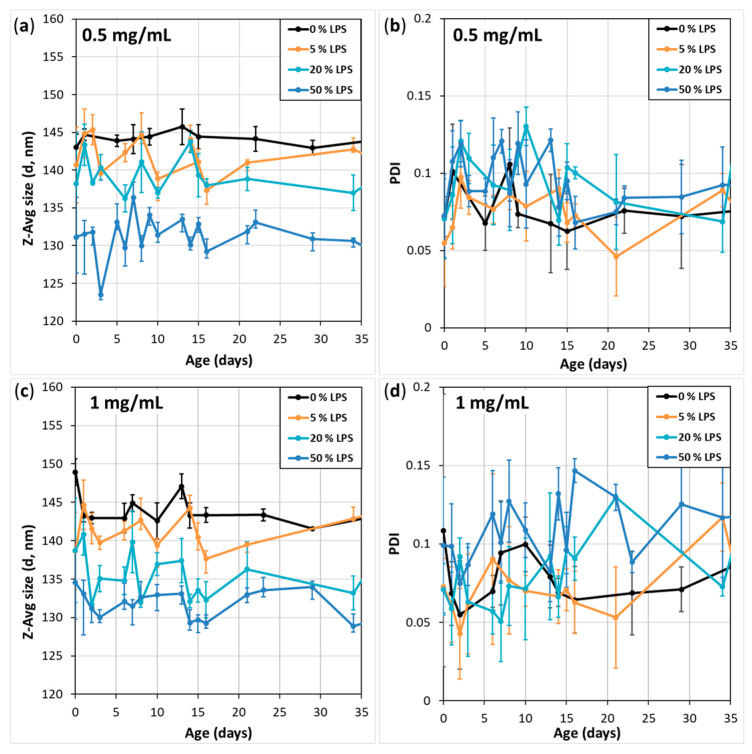
(**a**,**c**) Z-Avg size and (**b**,**d**) PDI stability of extruded DMPC-LPS vesicles with (**a**,**b**) 0.5 mg/mL and (**c**,**d**) 1 mg/mL total lipid concentration at 25 °C.

Samples of two different c_total_ values were followed, 0.5 mg/mL (Figure 3a,b) and 1 mg/mL (Figure 3c,d), according to our intention to use these instead of the more costly 2 mg/mL samples. The LPS contents of the samples were 0/5/20 and 50 *w*/*w*%. Figure 3a,c data lines represent the LPS content dependence of the vesicles’ Z-Avg size: the vesicles kept their original sizes. Additionally, PDI values (Figure 3b,d) remained around the initial value of 0.1, proving that the vesicles have good size stability.

### 3.3. Zeta Potential of the Vesicles

**Figure 4:**
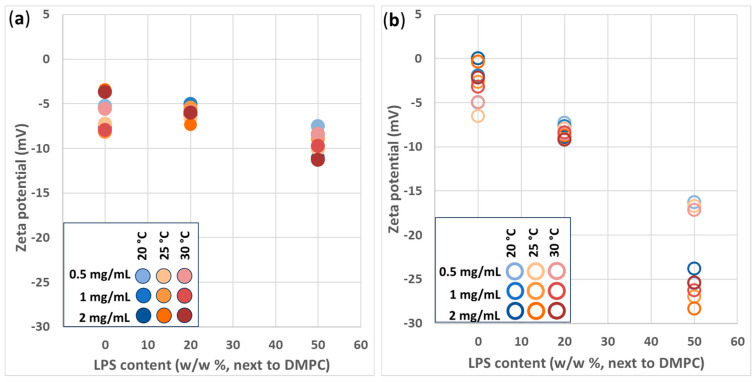
Zeta potential variation in (**a**) extruded and (**b**) hydrated DMPC-LPS vesicles with temperature (20–30 °C), LPS concentration (0–50 *w*/*w*%), and total lipid concentration (0.5–2 mg/mL) values.

Zeta potential correlates with the electrical potential of the slipping plane of the dispersed vesicles. The extruded and the hydrated samples are shown in Figure 4a and Figure 4b, respectively, with different LPS content, temperature, and c_total_. As can be seen in both figures, there is no evident or significant dependence of the zeta potential on temperature and c_total_. However, with increasing LPS content, the overall zeta potential becomes more negative for both extruded and hydrated samples. Extruded and hydrated DMPC vesicles with 0% LPS have slightly negative −5 mV and a 0–(−5) mV potential, respectively. A further shift of the zeta potential to more negative values with the addition of LPS was the following: with 20% LPS, the vesicles had ≤−5 mV for extruded and ~(−8) mV for hydrated samples; with 50% LPS, they had −10 mV for extruded and −25 mV for hydrated vesicles. The presence of negatively charged LPS is more expressed in the case of hydrated samples. The blending of the LPS and DMPC is poorer without proper mechanical agitation; thus, and furthermore, structures of LPS can also be formed spontaneously, representing their characteristic −20 to −30 mV zeta potential [36,37]. The size distributions in Figure S3 also suggest that the more LPS are introduced into the hydrated system, the more the smaller (~100 nm) structures are present, which can be LPS structures, as shown in Figure S3b.

Assessing stability from the zeta potential values would lead to the assumption that the extruded samples must be less stable (aggregation- and sedimentation-free) than the hydrated ones. In practice, however, the extruded vesicles proved to be aggregation-free and much more stable in terms of sedimentation. As the previous stability report (Figure 2) showed, even −5 mV was enough to keep the extruded vesicles free from aggregation. 

All the hydrated samples sedimented heavily in the time of half a day, seemingly ’ignoring’ the high negative zeta potential values, and even when re-dispersed, the samples were still cloudy. This can be explained by the presence of larger hydrated vesicles that are able to scatter light and be affected by gravity. DLS results on the relation of hydrated larger vesicles and smaller ones are discussed in the Supplementary Materials with the help of Figure S3a–d.

### 3.4. Spectral Features and Opacity

To our best knowledge, it is not usual to investigate LPS (and LPS-containing vesicles) without fluorescent labeling, but since, in practice, LPS may contain residual protein moieties (see the LPS Specification Sheet) [38], it seemed reasonable to execute this measurement.

**Figure 5:**
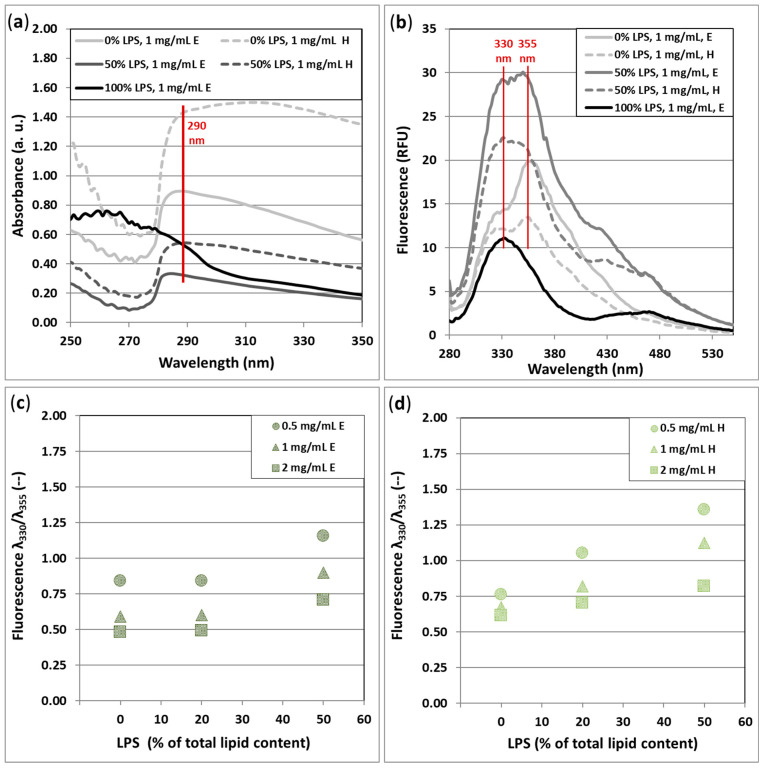
Absorbance and fluorescence aspects of LPS-DMPC samples. (**a**) Typical absorbance spectra of low- and high-concentration samples; (**b**) typical fluorescence spectra of samples with low and high LPS content, with 1 mg/mL total lipid concentration; (**c**,**d**) represents the ratio of two fluorescence peaks (330 nm and 355 nm) of the extruded and hydrated samples, respectively, as a function of the LPS content and the total lipid content, 0.5/1 and 2 mg/mL. Fluorescence data were collected after excitation at 260 nm, 25 °C. The indexes E and H in the insets mean extruded or hydrated vesicles, respectively.

The absorbance measurement shown in Figure 5a was meant only to assist the fluorescent scan. Below 270 nm, the spectra were intensively noisy, with no outstanding absorbance peak. From around 280 nm, starting at a peak with λ_max_ = 290, the spectra become smooth. Despite the presence of this peak, the excitation of the fluorescence was set to 260 nm (Figure 5b), since the lowest absorbance λ_max_ of the fluorescent amino acids is around 260 nm, which belongs to phenylalanine. Additionally, the 100% LPS-containing sample had a λ_max_ = 260 nm absorption peak (Figure 5a). In Figure 5b, the characteristic fluorescence of LPS-DMPC compositions is shown. The double peak between 280 and 410 nm, which partially covers the fluorescence region of the aromatic amino acids, can be separated into two peaks with λ_max_ = 330 nm and λ_max_ = 355 nm. Both peaks are present in every sample, even the solely DMPC-containing samples, which have no amino acid content, though DMPC has the emission features of phosphatidylcholine [39]. In the pure LPS-containing sample, the 330 nm peak largely dominates, and the 355 nm peak is stronger when DMPC is more abundant. Concerning the above findings, we showed that the ratio of the two fluorescent peaks’ maxima values correlates with the LPS ratio (% of the total lipid content) (Figure 5c,d). A clear correlation was found in the case of the hydrated samples (Figure 5d); however, despite the minimal increment between the 0% and 20% LPS samples, the phenomenon is noticeable in the case of extruded samples (Figure 5c) as well.

The fluorescence λ_330_/λ_355_ ratio of extruded samples is generally lower than that of the hydrated, which means that the relative fluorescence of LPS is lower when it is blended with and buried in DMPC more intensively by the extrusion.

**Figure 6:**
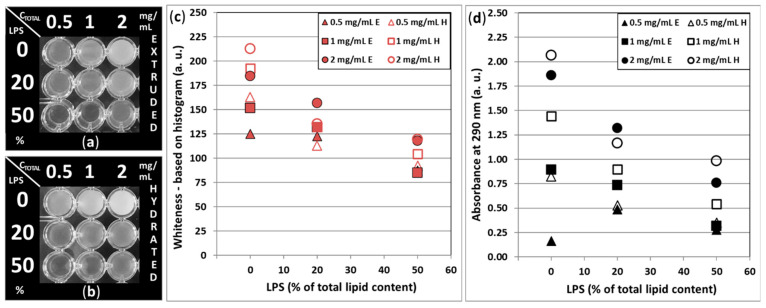
Representations of LPS-DMPC liposome-containing sample clarity at 25 °C. (**a**,**b**) Greyscale photographs of extruded (E) and hydrated (H) samples with different LPS content and total lipid content (c_total_), filled in optical plate wells; (**c**) color (white) coordinates (normalized to black→white: 0–255 bytes) sampled from a and b photographs, as a function of LPS content and total lipid content; (**d**) absorbance of the liposome samples at 290 nm.

The opacity of the LPS-DMPC samples was investigated via two methods, one digital photograph histogram analysis, and a spectral method based on the absorbance of the samples at 290 nm. In Figure 6a,b, the greyscale version of the photographs taken of the extruded and hydrated samples in photometric plates, respectively, are shown. The color (white) coordinates were recorded from each well and were plotted against the LPS content of the samples (Figure 6c), where a ‘0’ value means total black and a ‘255’ value refers to a completely white object. As can be seen by the naked eye, the opacity of the samples heavily depends on both c_total_ and the LPS content. Increased LPS content enhances the clarity of the samples, which is due to its size-lowering effect, as was demonstrated by size analysis, while the increasing c_total_ increases the opacity. Considering the effect of extrusion, it can be concluded that, in the case of low LPS content, extrusion decreases the vesicle size; thus, the amount of the highly scattering vesicles also decreases. With high LPS%, the effect diminishes, which also supports the size analysis report.

In Figure 6d, absorbance values recorded at 290 nm of the LPS-DMPC samples are shown, as a function of LPS content, c_total_, and extrusion. The resulting trends are very similar to that of the image analysis, with few deviations.

### 3.5. Density Measurements

The magnitude of the samples’ density values is defined by the solvent itself, since it is its most abundant component, and the vesicles are practically only curved 2D interfaces.

**Figure 7:**
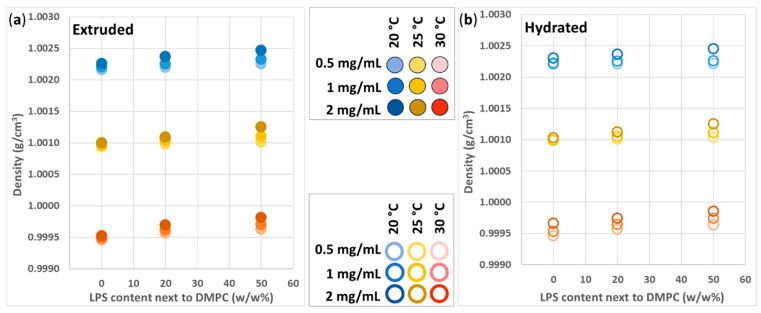
Density of (**a**) extruded and (**b**) hydrated DMPC-LPS samples with 0.5 (light), 1 (medium), and 2 mg/mL (dark shade) c_total_, at 20 (blue markers), 25 (yellow markers), and 30 °C (red markers).

Another crucial parameter is the temperature for the thermal expansion of the materials. The density of liposome-containing samples was clearly affected by temperature. At 20, 25, and 30 °C, the density varied around ~1.0023, ~1.0011, and ~0.9997 g/cm^3^, respectively (Figure 7a,b).

**Figure 8:**
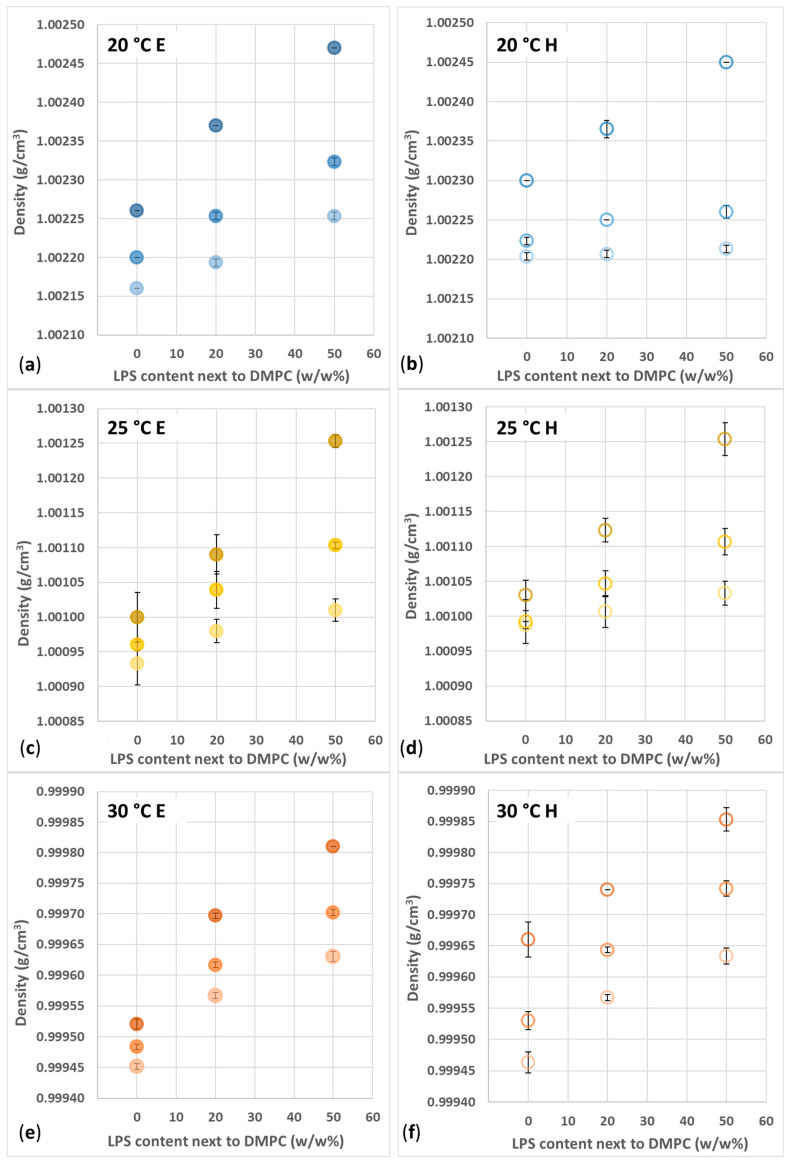
Density of (**a**,**c**,**e**) extruded (E) and (**b**,**d**,**f**) hydrated (H) DMPC-LPS vesicles with LPS concentration (0–50 *w*/*w*%), at temperatures of (**a**,**b**) 20 (blue), (**c**,**d**) 25 (yellow), and (**e**,**f**) 30 °C (orange) and with total lipid concentrations of 0.5 (light),1 (medium), and 2 mg/mL (dark shade). The results are mean ± SD obtained from 3 independent experiments in each series.

The minuscule differences between the various vesicle compositions can originate from several factors. The amount of hydrated water, denser than bulk water, around the head groups of the lipid molecules and negatively charged LPS compounds depends on the accessible number of headgroups in the sample. Thus, density is expected to increase upon increased LPS content and c_total_. The detailed representations of the density of extruded samples (Figure 8a,c,e) show that the increased LPS% and c_total_ result in higher density. In the case of the hydrated samples (Figure 8b,d,f), one can see similar trends.

With higher c_total_, the LPS% contribution is more pronounced, and the density–LPS% slopes are steeper. This phenomenon, i.e., the density–LPS% slope, is not just parallelly shifted from the 0.5 mg/mL values, as might be explained by the number of the vesicles being so high that they are closer to each other, and their neighboring hydration shells might entrap and coordinate/structurize some more water from the bulk phase, resulting in more hydration water compared to a more dilute system.

Comparing the extruded and hydrated samples, one can find some further differences. At 25 °C and 30 °C, all hydrated samples proved to be denser than the extruded ones. (Figure 8c–f). This behavior cannot be explained based on the already discussed factors (temperature, LPS%, and c_total_). Due to technical limitations, this study does not aim to reveal the phase structures of the vesicle and their macroscopic consequences; however, we aim to propose possible explanations for some non-trivial differences between the samples’ density values. There are two aspects that we need to consider. One is we already referred to with the data presented in Figure S3c,d, i.e., that the hydrated samples, due to the poor blending, tend to contain a significant number of separate LPS-rich structures, which have higher density compared to LPS-DMPC vesicles. This simply increases the average density of the sample. The second possible solution is in relation to the fluidity of the membrane. From other studies, it is known that, depending on the melting temperatures of the constituents, LPS tend to migrate within the hosting lipid ‘sea’, can form island-like domains, and can even detach from the host vesicle to form pure LPS vesicles [13,34]. In our case, the phase-transition temperature of DMPC is around 24 °C; hence, in the 25–30 °C range, the mobilized DMPC molecules can give space to migration, even detachment. Since DMPC mobility should be the same in the case of the hydrated and extruded samples as well, the main difference probably comes from the initial imperfect blending of the hydrated vesicles, with a possibly more domain-like structure, which finally allows more straightforward detachment upon heating. This phenomenon would also result in higher sample density.

Since, below 24 °C, there is a small chance for LPS migration, at 20 °C, the density data should reflect the initial (after-preparation) structure of the vesicles. The hydrated vesicles are multilamellar structures; i.e., several different-sized vesicles are encased in each other, and the probability of the interaction of their hydration shells (discussed in the c_total_-dependence paragraph) is higher compared to the case of unilamellar extruded vesicles. With 0% LPS, the proposed 3D provides higher density compared to the extruded samples. Upon the addition of 20 or 50% LPS, this hydration structure becomes corrupted by the LPS, lowering the overall density.

### 3.6. High-Resolution Ultrasound Spectroscopy—Velocity and Attenuation

**Figure 9:**
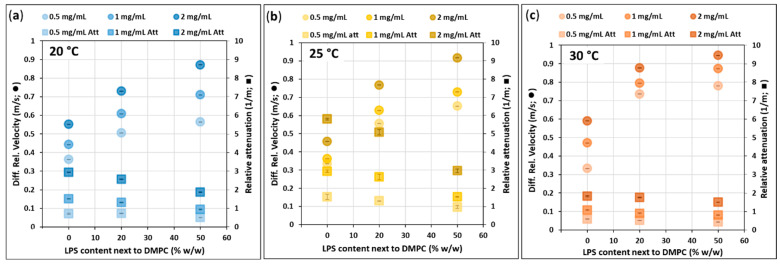
Differential relative velocity of ultrasound (circles) and its attenuation (squares) while propagating through the extruded DMPC-LPS samples measured at the frequency 15.035 MHz at the temperatures (**a**) 20, (**b**) 25, and (**c**) 30 °C and at the vesicle concentrations of 0.5 mg/mL (light), 1 mg/mL (medium), and 2 mg /mL (dark shade).

**Figure 10:**
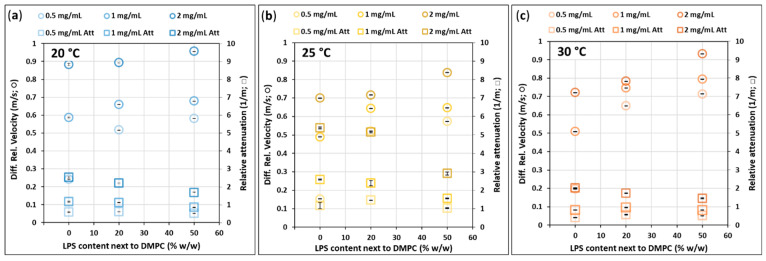
Differential relative velocity of ultrasound and its attenuation while propagating through the hydrated DMPC-LPS samples measured at the frequency 15.035 MHz at the temperatures (**a**) 20, (**b**) 25, and (**c**) 30 °C and at the vesicle concentrations of 0.5 mg/mL (light), 1 mg/mL (medium), and 2 mg /mL (dark shade).

In general, the differential relative velocity is proportional to the LPS content (added hydration water) and to the total lipid content, c_total_, in good agreement with the densitometry data but with more deviation from linear behavior (Figure 9 and Figure 10). Attenuation values are proportional to c_total_. However, attenuation is inversely proportional to LPS content, which can be explained by the decreasing vesicle sizes with LPS content (less scattering) and by the fact that LPS-rich domains are built up denser, mechanically more stable than those without LPS [13]. With temperature, the velocity increases in the samples, and the c_total_-dependence of velocity is lowered, while temperature has a lowering effect on attenuation.

At 25 °C (Figure 9b), the velocity values of samples with 0% LPS (0.5, 1, and 2 mg/mL) are lower than those at 20 and 30 °C (Figure 9a,c). Additionally, at 25 °C, the attenuation has a very strong c_total_-dependence, differing from both 20 and 30 °C measurements, and this stands for both extruded (Figure 9b) and hydrated (Figure 9b) samples. These deviations from the trends, experienced at 25 °C, are probably due to the proximity of the phase-transition temperature (*T_m_* ≈ 24 °C) of DMPC, where the building molecules have high conformational freedom [23], resulting in an expanded state.

HRUS measurements of the hydrated samples (Figure 10) came up with similar values and trends as the extruded ones. However, 0% LPS vesicles in the case of t_otal_ = 0.5 mg/mL and 2 mg/mL represent extremely low and high velocity values, respectively; the t_otal_-dependence of velocities is higher than any other samples. This deviation caused by the proposed multilamellar 3D hydration structure, discussed in Figure 8b, points out the significance of the extrusion.

At 20 °C, the velocity in t_otal_ = 2 mg/mL hydrated samples (Figure 10a) is generally higher than at 25 and 30 °C (Figure 10b,c), which contradicts the expectation that, in this temperature range, the sound velocity in water should increase with the temperature. Seemingly, the high t_otal_ induces a multilamellar structure, which is rich in hydration water and only mildly affected by the additional LPS.

### 3.7. Specific Adiabatic Compressibility of Vesicles

For further compressibility-related results, please see Figure S4 for partial specific volumes, Figures S5 and S6 for a detailed representation of specific adiabatic compressibility of extruded and hydrated vesicles, respectively, and Figure S7 for a comparison of hydrated and extruded samples by different t_otal_ values.

**Figure 11:**
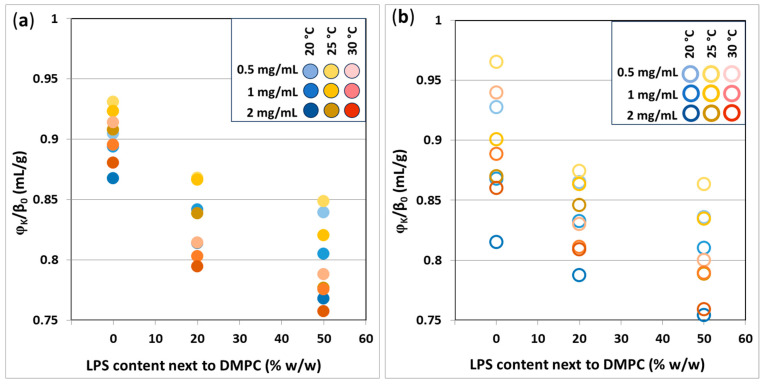
Specific adiabatic compressibility of (**a**) extruded and (**b**) hydrated DMPC-LPS vesicles (0.5, 1, and 2 mg/mL t_otal_) at 20, 25, and 30 °C.

From Figure 11, it can be concluded that the negative dependence of specific adiabatic compressibility on LPS% and t_otal_ is well expressed in both hydrated (Figure 11a) and extruded (Figure 11b) cases. Regarding the temperature, vesicles are provided with the highest compressibility at 25 °C, both hydrated and extruded (yellow markers in Figure 11). At 25 °C, slightly above the melting temperature (*T_m_*) of DMPC, the volume of the phospholipid molecules increases so that compressibility can reach a maximum around this temperature [28]. This explains the lower *w*_k_./*β*_0_ at either 20 °C or 30 °C. However, under or below *T_m_*, there is some variation in compressibility within the same LPS% sample groups; hence, from these measurements, a conclusive order cannot be defined by temperature.

A comparison of the extruded and hydrated samples shows that the adiabatic compressibility of hydrated vesicles has a wider distribution in all LPS% cases, most specifically with 0% LPS, where the hydrated data series spans the double compressibility range of the extruded ones. This is in good alignment with the velocimetry results.

## 4. Conclusions

The preparation of co-extruded *Salmonella enterica* so. Enteritidis LPS with DMPC was successful by means of good over-a-month size stability. In the wide range of applied LPS, it proved to be feasible to obtain and maintain stability and a narrow size distribution. LPS had relevant effects on the DMPC vesicles. A size-lowering effect was demonstrated via ‘shrinking’ pure DMPC vesicles from around 138 nm diameter to 133 and 125 nm diameter with 20% and 50% LPS, respectively. Being a negatively charged molecule, when added to DMPC, the LPS provided more negative charge, resulting in more negative zeta potential, from −5 mV down to −10 mV, which is usually not high enough to maintain such long stability; therefore, we must assume that other phenomenon, e.g., steric stabilization also takes place. The third important physical contribution of LPS to the vesicles is that they increase the density of the samples. Since this study does not consider direct information regarding the packaging structure of the lipid bilayer, as an explanation, we rely on the fact that the negative charge of LPS carries a hydrate shell, which is denser than bulk water. This is reflected by the higher density, higher sound velocity, and in accordance, the lowered relative attenuation and lower specific adiabatic compressibility of the samples of DMPC vesicles containing LPS as well.

Total lipid content, similarly, with respect to the LPS ratio, also increased the density and sound velocity and subsequently decreased the compressibility; however, in the attenuation values, we found an increment, probably caused by the higher number of vesicles causing more soundwave scattering. The total lipid content did not affect the size- and charge-related attributes very much.

The increased measurement temperature caused a 15–20 nm diameter increase when 20 °C and 30 °C were considered but did not affect the polydispersity index and the zeta potential of the samples. Sound velocity was increased, and the relative attenuation and specific adiabatic compressibility were lowered at 30 °C compared to 20 °C. However, at 25 °C, around the *T_m_* of DMPC, a definite irregularity occurred, causing a minimum in the sound velocity and a maximum in the attenuation and compressibility. This anomaly is the consequence of the phase change of DMPC, which gives the lipid molecules mobility, expanding their space while reducing macroscopic density.

The above-discussed results and conclusions stand only partially for the solely hydrated vesicles. The LPS ratio, here also, is a size-reducing factor, but the sizes are much higher than the extruded diameters. The PDI is around 1 with 0% LPS and a constant 0.6 with higher LPS contents. Regarding the zeta potential, at a lower LPS content, both vesicle structures display similar negative charges, but with 50% LPS content, the hydrated vesicles have a −25 mV zeta potential. This high value is probably due to the lack of good miscibility of LPS in DMPC and the lack of blending assist of the extrusion. The sample probably contains a significant population of vesicles consisting largely of LPS, with high negative potential. Density-related effects of LPS are like those expressed on extruded ones.

The total lipid content has no significant effect on hydrated samples, except for 0% LPS, where t_otal_ induces a diameter increase.

Unlike with extruded samples, temperature has no visible largening effect on hydrated vesicles. The anomalistic extremities at 25 °C are similar to those of the extruded vesicles in the case of sound velocity, relative attenuation, and specific adiabatic compressibility. However, the trends of the above attributes in the 20–30 °C range are less obvious.

From the practical point of view, the high-level integration of *S. enterica* so. Enteritidis LPS into DMPC vesicle bilayers requires extrusion. Otherwise, the (uni)lamellarity of the vesicles is at least questionable, and consequently, the quantity of LPS on the vesicles’ surfaces stays undetermined. Therefore, the quantification of an LPS-specific interaction at the vesicle/buffer interface is impossible. Moreover, the solely LPS-containing aggregates and vesicles, which remain in the hydrated samples, have packed LPS with sterically different positions, compared with those distributed on the surface of DMPC vesicles, and this is very likely to affect their molecular accessibility.

In the meantime, the extruded LPS-DMPC vesicles proved to be stable and uniform, with an ideally low polydispersity index. The trustworthily variable range of the total lipid concentration, LPS ratio, and temperature, and the fact that the extruded samples withstood numerous heating, stirring, degassing, and measuring procedures without budding or fission, makes these vesicles more than promising subjects to further research in the field of biorecognition, either applied in liquid media or transformed into sensing surfaces.

## Data Availability

Data will be made available on request.

## Supplementary Materials

The following supplementary information can be downloaded at: https://www.mdpi.com/article/10.3390/biomimetics10010055/s1, Figure S1: PDI variation in extruded LPS-DMPC vesicles with temperature (20–30 °C), LPS concentration (0–50 *w*/*w*%) and total lipid concentration (0.5–2 mg/mL); Figure S2: PDI variation in hydrated DMPC-LPS vesicles with temperature (20–30 °C), LPS concentration (0–50 *w*/*w*%) and total lipid concentration (0.5–2 mg/mL); Figure S3: Comparisons of typical intensity- (light color), volume- (medium color) and number-related (dark color) DLS size distribution patterns of hydrated (blue dashed line) and extruded (red solid line) LPS-DMPC liposomes with different LPS content: (a) 0%, (b) 100%, (c) 20%, and (d) 50%; Figure S4: Partial specific volume of (a) extruded and (b) hydrated DMPC-LPS vesicles (0.5, 1, and 2 mg/mL t_otal_) at 20, 25, and 30 °C; Figure S5: Specific adiabatic compressibility of extruded LPS-DMPC liposomes with different LPS content and t_otal_ at (a) 20 °C, (b) 25 °C, and (c) 30 °C; Figure S6: Specific adiabatic compressibility of hydrated LPS-DMPC liposomes with different LPS content and t_otal_ at (a) 20 °C, (b) 25 °C, and (c) 30 °C; Figure S7: Comparison of specific adiabatic compressibility of hydrated and extruded LPS-DMPC liposomes with different LPS content and temperatures at (a) t_otal_ = 0.5 mg/mL, (b) t_otal_ = 1 mg/mL, and (c) t_otal_ = 2 mg/mL [40,41].
