# Supplementary material for: The Effect of Lipopolysaccharides from Salmonella enterica on the Size, Density, and Compressibility of Phospholipid Vesicles
Source: Biomimetics (Basel). 2025 Jan 15;10(1):55. doi: 10.3390/biomimetics10010055 (PMC11759865; doi:10.3390/biomimetics10010055)
Supplement: Supplementary file 1 [file biomimetics-10-00055-s001.zip › biomimetics-3326977-supplementary.pdf]

The Effect of Lipopolysaccharides from Salmonella enterica on the Size, Density, and Compressibility of Phospholipid Vesicles

Tamás Szabó 1,* , Zuzana Garaiová 2, Sopio Melikishvili 3, Marek Tatarko 2, Zsófia Keresztes 1 and Tibor Hianik 2,*

1 Functional Interfaces Research Group, Institute of Materials and Environmental Chemistry, HUN-REN Research Centre for Natural Sciences, Magyar Tudósok Körútja 2, 1117 Budapest, Hungary; keresztes.zsofia@ttk.hu

2 Department of Nuclear Physics and Biophysics, Faculty of Mathematics, Physics and Informatics, Comenius University, Mlynska Dolina F1, 84248 Bratislava, Slovakia; zuzana.garaiova@fmph.uniba.sk (Z.G.); marek.tatarko@fmph.uniba.sk (M.T.)

3 Institute of Medical Physics and Biophysics, Faculty of Medicine, Comenius University, Sasinkova 2, 81372 Bratislava, Slovakia; sopio.melikishvili@fmed.uniba.sk

* Correspondence: szabo.84.tamas@ttk.hu (T.S.); tibor.hianik@fmph.uniba.sk (T.H.)

Supplementary Material

S1. Polydispersity Index of the Vesicles Modified by Lipopolysaccharides

Polydisperstiy index (PDI) is the indicator of the narrowness of the size distribution. It is calculated automatically by the software from the intensity data as well (PDI = [(St.dev^2)/(Mean^2]). Variation of PDI for LUV contained LPS is presented on Figure S1 and those for hydrated liposomes is on Figure S2.

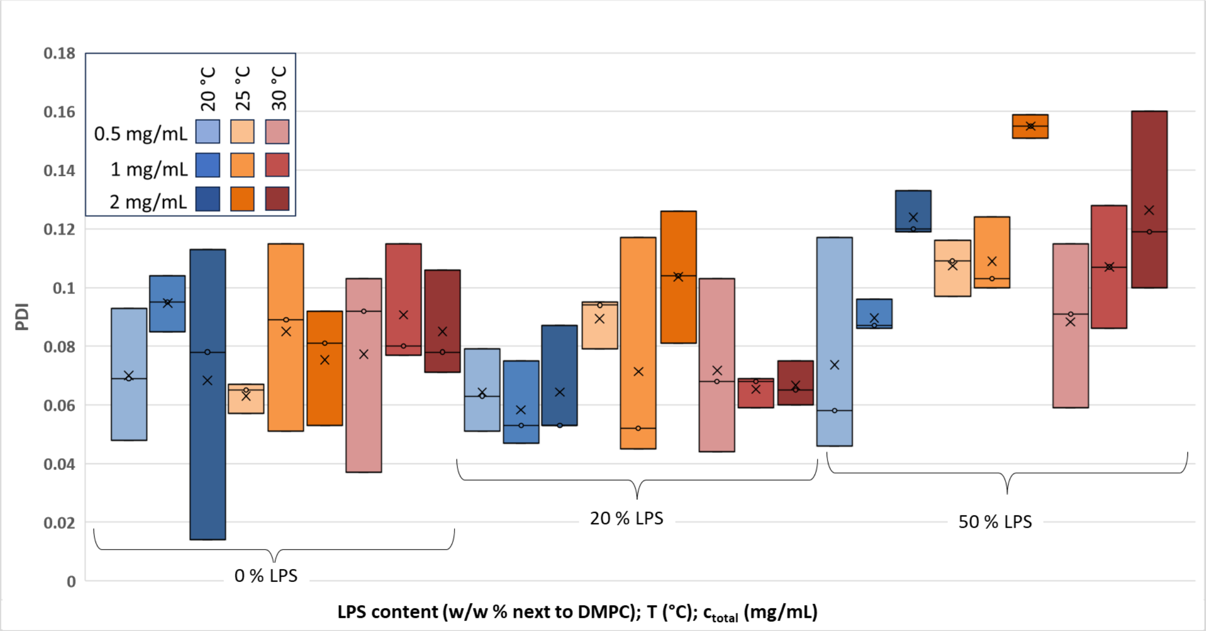

Figure S1. PDI variation of extruded LPS-DMPC vesicles with temperature (20-30 °C), LPS concentration (0-50 w/w%) and total lipid concentration, c_{total} (0.5-2 mg/mL).

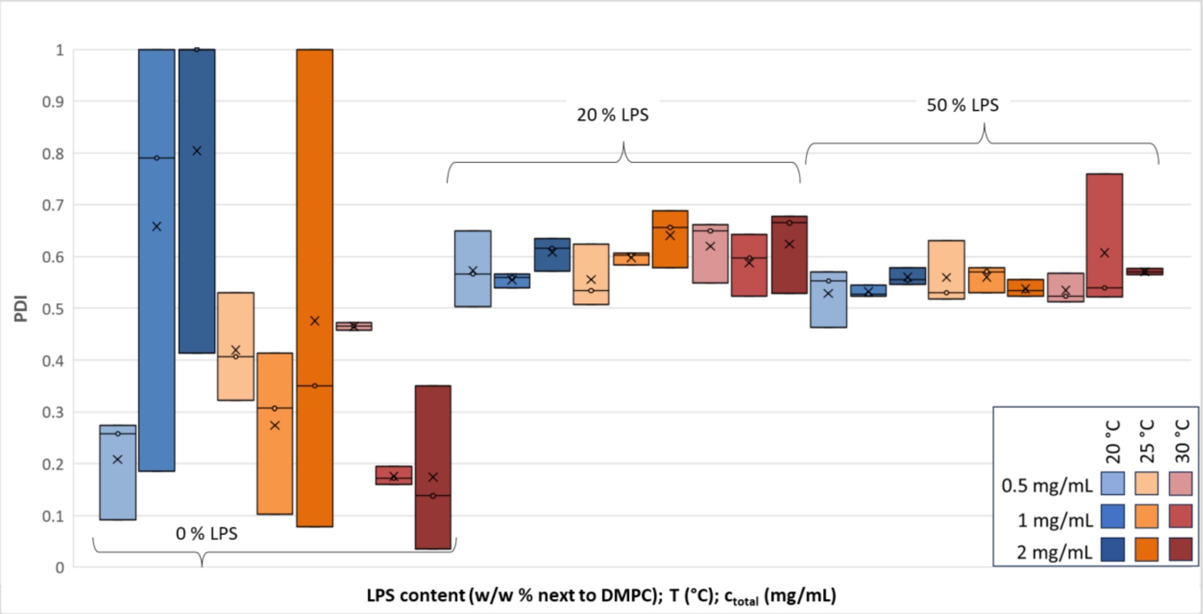

Figure S2. PDI variation of hydrated DMPC-LPS vesicles with temperature (20-30 °C), LPS concentration (0-50 w/w%) and total lipid concentration, c_{total} (0.5-2 mg/mL).

S2. Comparison of Intensity, Volume and Number-Based Size Distributions of LPS, DMPC and LPS-DMPC Structures

Figure S3 a-d illustrate the typical formation of vesicle-like structures with and without extrusion, in the absence or presence of LPS next to DMPC, at 20 °C, and regardless of the c_{total}. All the diagrams contain the size distribution, recorded by DLS, of the hydrated (dashed blue lines) and the extruded (continuous red lines) form of a typical sample, with three different measurement evaluation aspect: distribution by intensity, by volume and by number (pale, medium and darker shades, respectively). The point of this comparison is to show that it is not sufficient to use only one representation of these samples for a thorough description. In Figure S3a the pure DMPC is shown, where without extrusion, only large (giant) vesicles are present with wide distribution, and after extrusion all the large structures are squeezed into the ~100 nm size. It is important to note that all three evaluations are in good agreement, except for some differences in the 100 nm region. Usually, a certain difference is present between the results of the three evaluations. Therefore, we can conclude that DMPC by itself forms only giant vesicles when only hydrated.

In Figure S3b the pure LPS is presented, without any DMPC. From the position of dashed blue lines, it can be seen that a simply hydrated LPS forms much smaller structures (at this point it is unknown whether these are vesicles or not) than DMPC (Figure S3a). The different evaluation approaches show a 50 nm to 90 nm (by number and by intensity, respectively) diameter range. After extrusion one cannot see significant changes, which is understandable since the pore size of the filter is 100 nm.

Moving on to the mixtures of LPS and DMPC in Figure S3c one can see that the hydrated mixture, by the intensity and volume distribution, is shown to be also a mixture of larger (~1000 nm) and smaller (~100 nm) vesicles. However, the numerical distribution shows only the ~100 nm peak. This apparent contradiction can be resolved when we consider that intensity- and volume-related data evaluation is highly sensitive to larger particles, much over a proportional extent [1,2]. Knowing that the number-related result does not show larger particles, one can assume that indeed there are some large particles, but these, in number, do not contribute to the average size of the multitude of ~100 nm vesicles. Based on the above results we can propose that the simple addition (and obviously the incubated shaking period) of 20% LPS hinders the tendency of DMPC to form giant vesicles. After extrusion, the size remains around 100 nm.

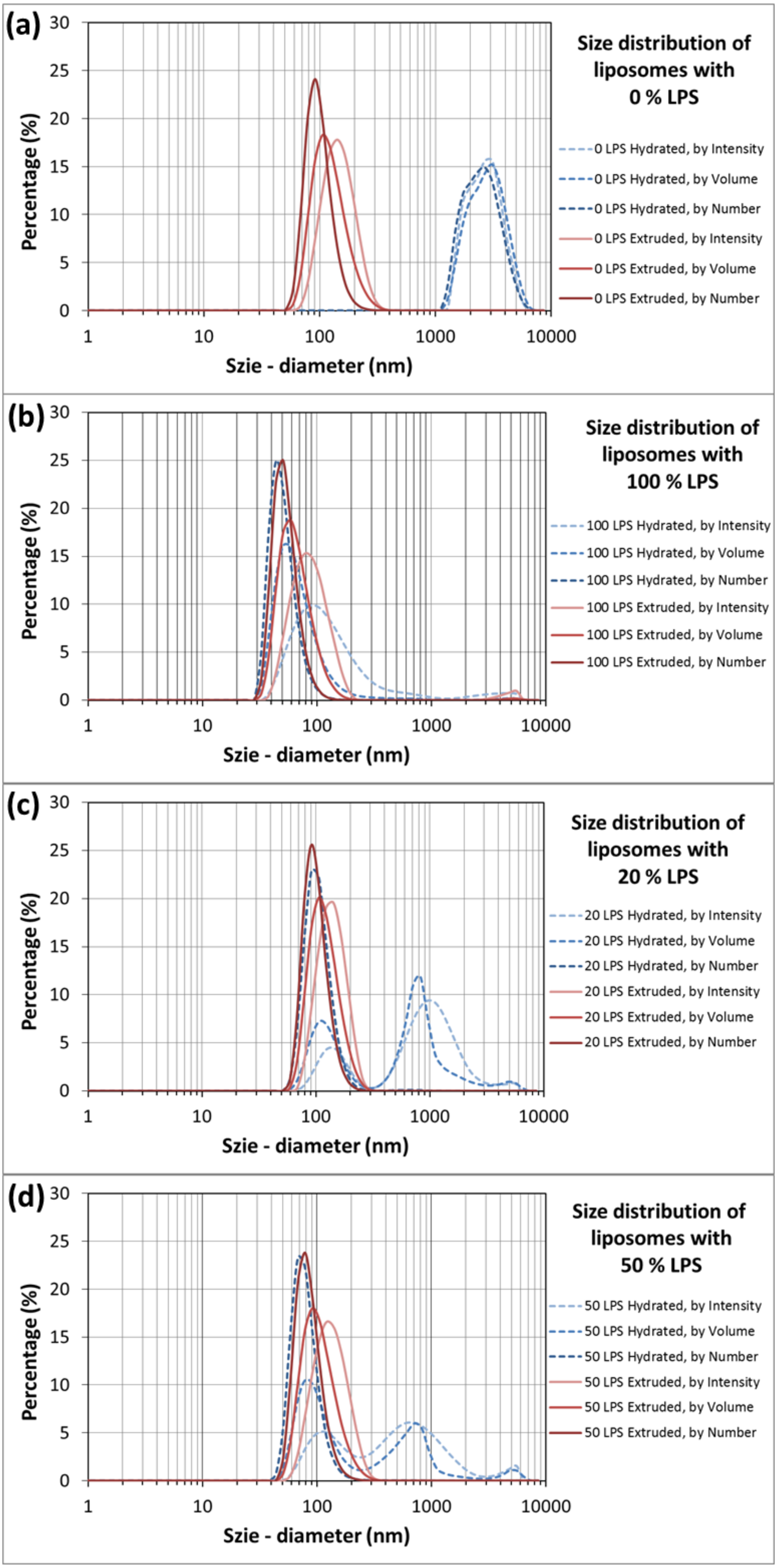
(a)
Size distribution of liposomes with 0 % LPS
0 LPS Hydrated, by Intensity
0 LPS Hydrated, by Volume
0 LPS Hydrated, by Number
0 LPS Extruded, by Intensity
0 LPS Extruded, by Volume
0 LPS Extruded, by Number
Percentage (%)
Szie - diameter (nm)
(b)
Size distribution of liposomes with 100 % LPS
100 LPS Hydrated, by Intensity
100 LPS Hydrated, by Volume
100 LPS Hydrated, by Number
100 LPS Extruded, by Intensity
100 LPS Extruded, by Volume
100 LPS Extruded, by Number
(c)
Size distribution of liposomes with 20 % LPS
20 LPS Hydrated, by Intensity
20 LPS Hydrated, by Volume
20 LPS Hydrated, by Number
20 LPS Extruded, by Intensity
20 LPS Extruded, by Volume
20 LPS Extruded, by Number
(d)
Size distribution of liposomes with 50 % LPS
50 LPS Hydrated, by Intensity
50 LPS Hydrated, by Volume
50 LPS Hydrated, by Number
50 LPS Extruded, by Intensity
50 LPS Extruded, by Volume
50 LPS Extruded, by Number

Figure S3. Comparisons of typical intensity- (light colour), volume- (medium colour) and number-related (dark colour) DLS size distribution patterns of hydrated (blue dashed line) and extruded (red solid line) LPS-DMPC liposomes with different LPS content: (a) 0%; (b) 100% ; (c) 20% and (d) 50%.

The above discussed phenomenon is shown very similarly in Figure S3d, with 50% LPS added to the sample. The only clear difference is that in the intensity- and volumetric representation the ratio of ~700 nm and ~100 nm vesicles is lower, compared to the 20% LPS sample, which could also confirm the presence of higher amount of LPS. Again, the numeric evaluation underlines that there are no significant number of giant structures in the sample.

The summary of this presentation firstly is that LPS by itself is also able to form uniform <100 nm structures, and secondly, the LPS can involve the DMPC into forming mutual liposomal structures even without extrusion. This latter does not mean that the extrusion is unnecessary, because the shear forces under extrusion probably exert more drastic effect on the mixing/blending of the two components.

In the light of the above discussed phenomena, referring back to the results of hydrated samples we can say that both Z-Avg - (Figure 2), and PDI values (Figure S2) of LPS-containing samples are lowered or narrowed compared to 0% LPS samples, because both values are calculated also on the basis of the intensity data which contains the >700 nm peak as well. If only the number-related evaluation were considered, the hydrated Z-Avg and PDI values would be much more similar i.e. lower to those of the extruded ones.

S3. Detailed Exhibition of Compressibility-Related Data: Partial Specific Volumes and Comparison of Specific Adiabatic Compressibility by Temperature and Total Lipid Content

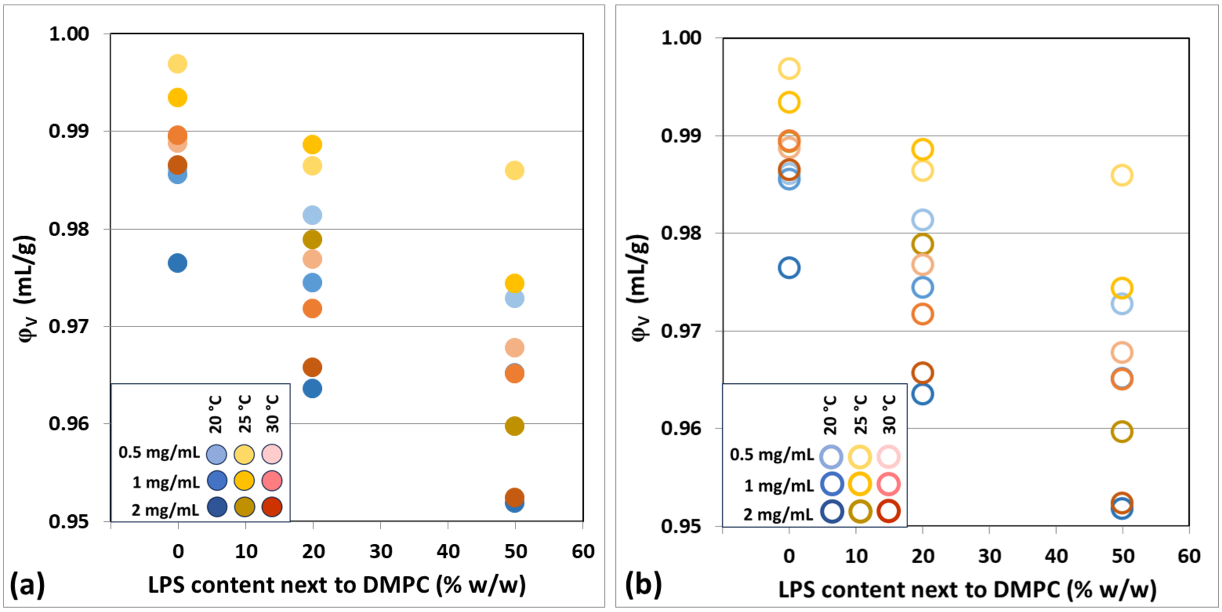

Figure S4. Partial specific volume of (a) extruded and (b) hydrated DMPC-LPS vesicles (0.5, 1 and 2 mg/mL c_{total}) at 20, 25 and 30 °C.

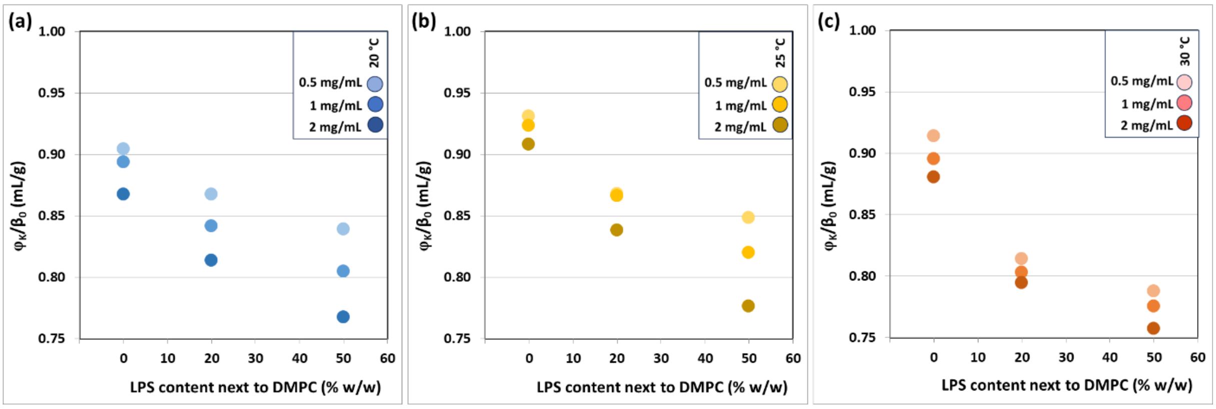

Figure S5. Specific adiabatic compressibility of extruded LPS-DMPC liposomes with different LPS-content and ctotal at (a) 20 °C; (b) 25 °C; and (c) 30 °C.

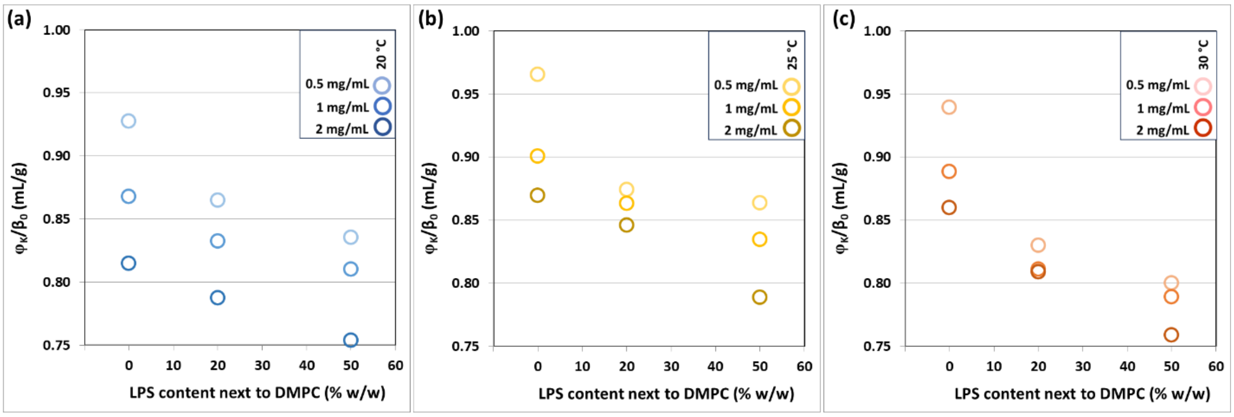

Figure S6. Specific adiabatic compressibility of hydrated LPS-DMPC liposomes with different LPS-content and c_{total} at (a) 20 °C, (b) 25 °C, and (c) 30 °C.

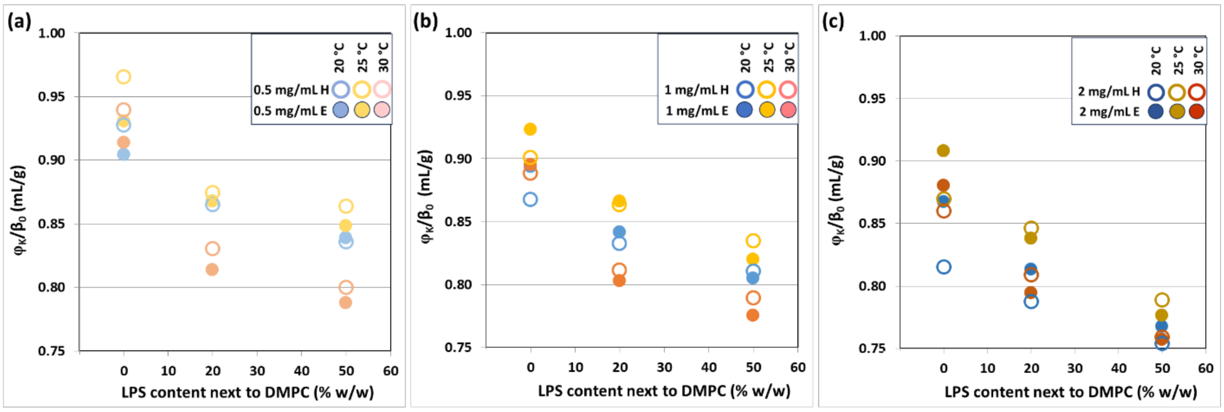

Figure S7. Comparison of specific adiabatic compressibility of hydrated and extruded LPS-DMPC liposomes with different LPS-content and temperatures at (a) c_{total} = 0.5 mg/mL, (b) c_{total} = 1 mg/mL, and (c) c_{total} = 2 mg/mL.

References

1. https://www.materials-talks.com/intensity-volume-number-which-size-is-correct/ (Last visited on 29.10.2024)
2. Malvern Zeta Sizer Nano Series user manual, 2013, Malvern Instruments Ltd., Enigma Business Park, Grovewood Road, Malvern, Worcestershire WR14 1XZ United Kingdom, page 11-15 Intensity, volume and number distributions in Chapter 11 on Size Theory.
